# Supplementary material for: Raman-free fibered photon-pair source
Source: Sci Rep. 2020 Feb 3;10:1650. doi: 10.1038/s41598-020-58229-7 (PMC6997395; doi:10.1038/s41598-020-58229-7)
Supplement: Supplementary file 1 — Supplementary Information. [file 41598_2020_58229_MOESM1_ESM.pdf]

Raman-free fibered photon-pair source

Supplementary Information

Martin Cordier,[1] Philippe Delaye,[2] Frédéric Gérôme,[3] Fetah Benabid,[3] and Isabelle Zaquine[1]

[1] *LTCI, Télécom Paris, Institut Polytechnique de Paris, 75013 Paris, France*
[2] *Laboratoire Charles Fabry, Institut d'Optique Graduate School, CNRS, Université Paris-Saclay, 91127 Palaiseau cedex, France*
[3] *GPPMM Group, XLIM Research Institute, CNRS UMR 7252, Université de Limoges, Limoges, France*

1/ Fiber design

The fiber is a tubular hollow-core photonic crystal fiber composed of 8 silica tubes with strut thickness $t = 630 \pm 20$ nm and with a length of $L = 1$ meter. Its 22 ± 1 μm effective core radius offers a good trade-off between losses, generation efficiency and modal content [1]. The fiber cross-section is shown in Fig. 1 a.
The guidance mechanism called inhibited-coupling relies on a coupling inhibition between guiding core modes and cladding modes (these fibers are also sometimes associated to the ARROW guidance mechanism: anti-resonance reflecting optical waveguide or anti-resonant hollow core fibers, for more details please refer to [2]). This is in contrast with the photonic bandgap guidance mechanism which relies on the existence of a spectral range void of cladding modes.

a. Dispersion: Simulations and experimental observations have shown that the dispersion properties of inhibited-coupling HCPCF can be well approximated by a "tube-type" model [1, 3, 4] where the HE$_{m,n}$ modes is described by:

$$n_{\text{eff}} = n_{gas} - \frac{j^2_{m-1,n}}{2k_0^2 n_{gas} R^2} - \frac{j^2_{m-1,n}}{k_0^3 n_{gas}^2 R^3} \cdot \frac{\cot\left[\Psi(t)\right]}{\sqrt{\epsilon - 1}} \cdot \frac{\epsilon + 1}{2}, \tag{1}$$

with : $\Psi(t) = k_0\, t\sqrt{n_{si}^2 - n_{gas}^2}$, R the fiber radius, $\epsilon = n_{si}^2/n_{gas}^2$, $j_{m,n}$ the nth root of the mth Bessel function J, t the silica strut thickness and n_{si} the silica glass refractive index. The slowly varying contribution of the effective index is described by the first two terms of Eq. (1) depending on gas dispersion and fiber core radius only. The third term introduces discontinuities in the dispersion as a result of resonances with the silica struts. The positions of these narrow non-guiding regions depend on silica strut thickness t and correspond to $\Psi(t) = l\pi$ with l integer. This defines a set of wavelengths $\lambda_l = \frac{2t}{l}\sqrt{n_{si}^2 - n_{gas}^2}$ where the fiber dispersion is divergent.

The simulated dispersion and the inverse group velocity ($\beta_1(\omega) = \frac{1}{v_g(\omega)}$) of the fiber are shown in Fig. 2. The fiber design was chosen to operate at convenient wavelengths. Indeed, the pump wavelength is set at 1033 nm which is commercially very common and the idler wavelength lies at telecom wavelength range, while the signal

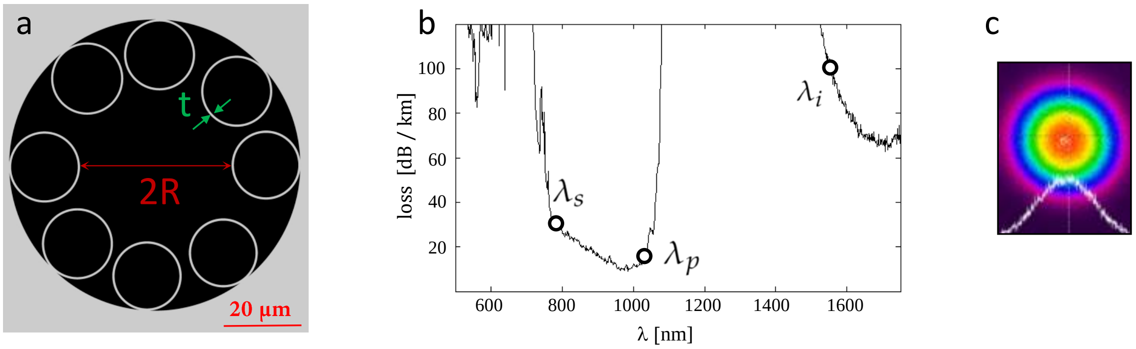

Fig. 1 **(a)** Optical image of the fiber cross-section. **(b)** Measured fiber transmission loss. **(c)** Measured spatial profile at the fiber output at pump frequency. In the spatial domain, the fiber can be slightly multimode depending on the coupling condition. At the maximum coupling efficiency, it is mostly the fundamental mode which is excited.

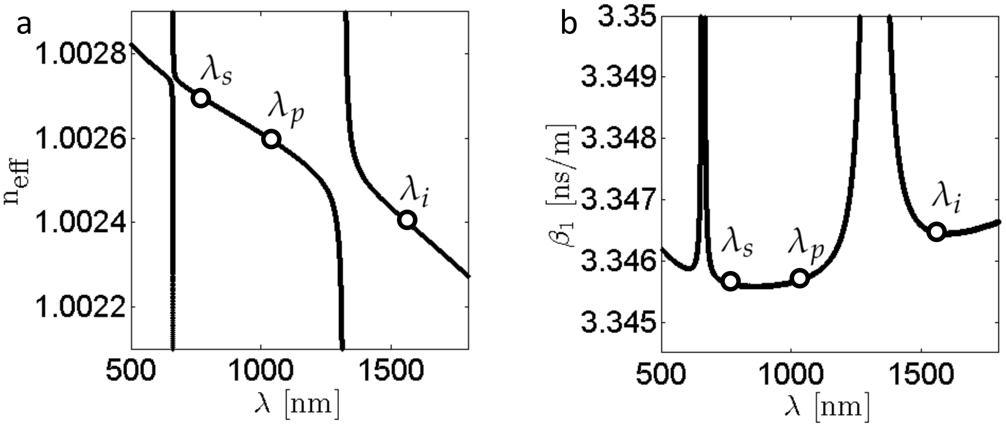

Fig. 2 **(a)** Simulated dispersion and **(b)** inverse group-velocity as a function of wavelength of the fiber when filled with xenon.

wavelength is in the range of atomic transitions and Silicon single photon detectors. Moreover, we also chose a design allowing to access a multiband FWM (i.e the pump and idler photon are separated by a discontinuity). As we have shown in [1, 5], such configuration allows to engineer the spectral correlations. The chosen fiber fulfils the "asymetric group-velocity matching condition": $\beta_1(\omega_p) \approx \beta_1(\omega_s)$ which is a condition to have spectrally factorable photon-pair [6].

b. Gas: In addition to the fiber geometrical design, gas temperature and, more notably, pressure can be used to actively impact the dispersion and consequently the phase-matching condition (see Eq. (1)). The refractive index of the gas at temperature T and pressure P_{gas} can be extrapolated from the general Sellmeier equation at standard temperature and pressure conditions (T_0,P_0) using:

$$n_{\text{gas}} \approx \sqrt{1 + (n_{\text{gas}}^2(\lambda, P_0, T_0) - 1).\frac{P_{\text{gas}}}{P_0}.\frac{T_0}{T}} \tag{2}$$

This equation shows that, pressure and temperature have similar influences but with opposite directions. Increasing the pressure/decreasing the temperature makes the gas more dispersive. The position of the mean zero-dispersion wavelength can be shifted with pressure or temperature modification (see Fig. 3). More precisely, as pressure increases, the signal and idler fulfilling the phase matching condition are further and further apart from the pump central frequency [5]. We used this feature to actively control the frequencies of the generated photons.

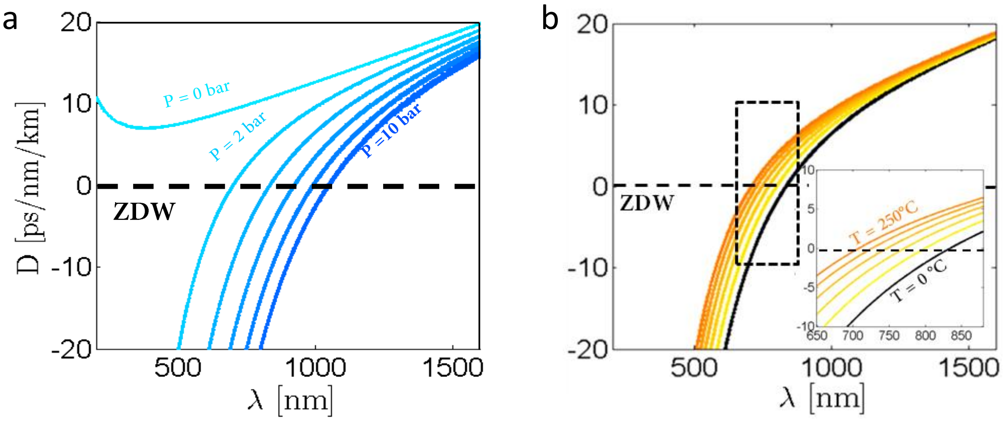

Fig. 3 **(a)** Group velocity dispersion (D $= -\frac{2\pi c}{\lambda^2}\frac{d\beta}{d\omega^2}$) at different pressures of xenon in a fiber of 22 μm at $T = 273$ K. **(b)** Same fiber design with a fixed pressure of 4 bar of xenon and for different temperatures. For clarity, the effect of the resonances (last term of Eq (1) is neglected.)

c. Nonlinearity and generation efficiency: When choosing a set of pump, signal and idler wavelength configuration, there exists many different fiber designs fulfilling the required phase-matching condition. Each of them are

defined by both fiber (radius, silica strut thickness, fiber length) and gas (temperature, pressure) parameters. For a given fiber radius and silica strut thickness, there always exists a certain pressure for which the phase-matching is obtained. Interestingly, the main tendency is that the lower the radius, the higher the gas pressure required to fulfil the phase-matching condition (see for instance Fig. 4). Thus, choosing a fiber with a small radius as a double

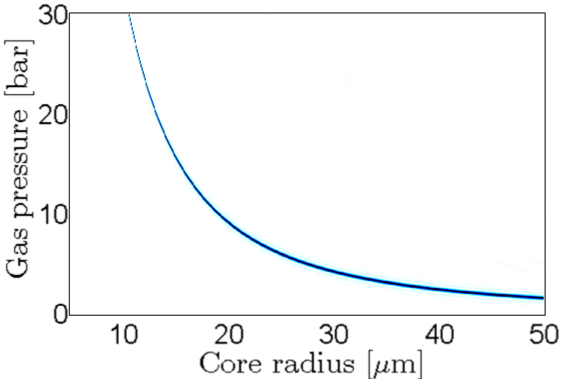

Fig. 4 Combination of radius and gas pressure (xenon) allowing to obtain a phase-matching with the configuration of wavelength ($\lambda_p \approx 1033$ nm, $\lambda_s \approx 770$ nm, $\lambda_i \approx 1570$ nm). The simulations are done with a fixed silica strut thickness parameter $t = 1250$ nm.

beneficial effect on the nonlinearity, firstly because the effective area becomes smaller and secondly because the required gas-pressure becomes higher, increasing even more the nonlinearity. Indeed, one can show that the number of generated pair is proportional to the square of the gas nonlinearity divided by the effective area [5]:

$$N_{\text{pair}} \propto \left(\frac{n_{2,\text{gas}}}{A_{\text{eff}}}\right)^2 \propto \frac{P_{\text{gas}}^2}{R^4} \tag{3}$$

Thus our current design ($L = 1$ m, $R = 22$ μm, $P_{\text{gas}} = 4$ bar, $t = 630$ nm) that exhibits a generation photon-pair rate of the order of $\eta \approx 1$ kHz at 20 mW of pump power, could be improved by 4 orders of magnitude when replaced by ($L = 10$ m, $R = 10$ μm, $P_{\text{gas}} = 30$ bar, $t = 1250$ nm), meaning an improved generation photon-pair rate of $\eta \approx$ 10 MHz is realisable, at the same pump power and repetition rate. It is noteworthy that several works have already demonstrated HCPCF filled with more than 70 bar of xenon [7, 8], demonstrating the feasibility of such example of new design.

d. Phase-matching: Using Eq. (1) and Eq. (2), one can simulate the configuration of pump, signal and idler wavelengths that fulfils the phase-matching condition ($\text{sinc}\big((2\beta(\omega_p) - \beta(\omega_s) - \beta(\omega_i)\frac{L}{2}\big) = 1$). For our fiber, the computed spectral density map is shown in Fig. 5. The color indicates the expected angle of the phase-matching ($\theta = -\arctan(\frac{\beta_{1p}-\beta_{1s}}{\beta_{1p}-\beta_{1i}})$. There exists three distinctive phase-matching regions corresponding to either singleband four-wave mixing (S) or multiband four-wave mixing (M1 and M2).

The singleband FWM generates the photon-pair more closer to the pump frequency. As the phase-matching angle $\theta \approx -45°$, in that configuration, the photon-pairs are highly spectrally correlated. On the contrary, the two multiband FWM allow the generation of factorable photon-pair ($\theta = 0°$, 45°, 90°) and with a very large detuning from the pump (more details in [1]).

e. Propagation Loss: As shown in Fig. 1.b. losses are of the order of $\approx$ 10-100 dB/km inside a given transmission band. These loss are quite negligible considering the one meter length of the fiber. However, loss increase rapidly as the wavelength approaches a band-edge. Therefore, it is important that the involved wavelengths λ_p, λ_s, λ_i are not too close to a discontinuity in order to enable efficient four-wave mixing.

2/ Measurement analysis:

a. Fit parameters: The exact polynomial fits parameters of the counts and histogram analysis are given in Table I. It is obtained by orthogonal distance regression method and takes into account the errorbar of each measurements. The fit are conditioned such that each parameters must be positive in order to have a proper physical interpretation. All terms related to Raman-scattering are either null or with a value below their errorbar.

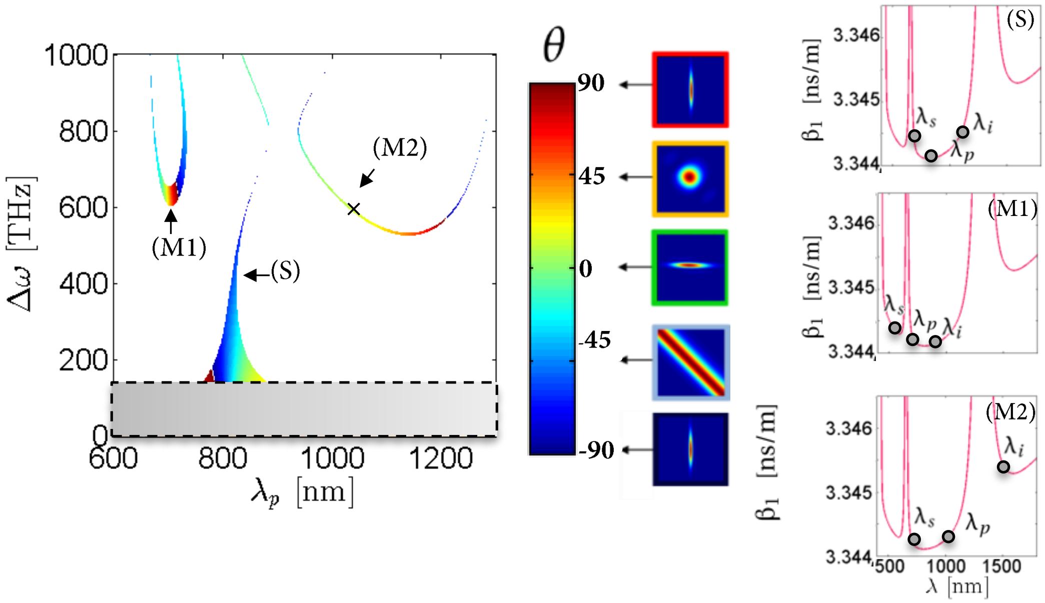

Fig. 5 Left: Simulation of the FWM spectral density map. The parameters used are the one of our fiber. The y-axis corresponds to the gap between pump and signal/idler frequencies $\Delta\omega = |\omega_p - \omega_{s/i}|$. (S) correspond to a singleband FWM whereas (M1) and (M2) correspond to a multiband FWM. The black cross describes our operating point($\lambda_p = 1033$ nm). The grey region corresponds to FWM where signal and idler are generated too close to the pump wavelength. Note that the lines have been thickened for improved visibility. Right: Corresponding group velocity relations.

Tab. I: Exact polynomial fit parameters obtained using orthogonal distance regression method.

Events	Fit	Coefficient
Counts analysis		
Counts Signal	$a + b\,P + c\,P^2$	$a = 313.26 \pm 34.9$ s^{-1}<br>$b = 0 \pm 2.11$ s^{-1} mW^{-1}<br>$c = 0.66248 \pm 0.0198$ s^{-1} mW^{-2}
Counts Idler	$a + b\,P + c\,P^2$	$a = 2120 \pm 14.9$ s^{-1}<br>$b = 0 \pm 1.01$ s^{-1} mW^{-1}<br>$c = 0.27109 \pm 0.0103$ s^{-1} mW^{-2}
Histogram analysis		
Coincidences	$a + b\,P + c\,P^2 + d\,P^3$	$a = 0 \pm 1.7$ s^{-1}<br>$b = 0 \pm 0.232$ s^{-1} mW^{-1}<br>$c = 0.031145 \pm 0.00623$ s^{-1} mW^{-2}<br>$d = 1.0291.\,10^{-5} \pm 3.94.\,10^{-5}$ s^{-1} mW^{-3}
Accidentals	$a + b\,P + c\,P^2 + d\,P^3 + e\,P^4$	$a = 0.0024451 \pm 0.00809$ s^{-1}<br>$b = 0 \pm 0.00154$ s^{-1} mW^{-1}<br>$c = 0 \pm 9.49.\,10^{-5}$ s^{-1} mW^{-2}<br>$d = 0 \pm 2.\,10^{-6}$ s^{-1} mW^{-3}<br>$e = 7.8281\,10^{-8} \pm 1.2\,10^{-8}$ s^{-1} mW^{-4}
Uncorrelated	$a + b\,P + c\,P^2$	$a = 0.00076152 \pm 0.000843$ s^{-1}<br>$b = 6.4151\,10^{-6} \pm 5.64\,10^{-5}$ s^{-1} mW^{-1}<br>$c = 2.4538\,10^{-6} \pm 5.18\,10^{-7}$ s^{-1} mW^{-2}

b. **Coincidence to accidental ratio (CAR)**: In our source, due to the negligible amount of Raman-scattering, the CAR is simply described by:

$$\mathrm{CAR} = \frac{N_{\mathrm{coinc}}}{N_{\mathrm{acc}}} \approx \frac{N_{\mathrm{pair}} + N_{\text{param-DC}} + N_{\text{DC-DC}}}{N_{\text{param-param}} + N_{\text{param-DC}} + N_{\text{DC-DC}}} \qquad (4)$$

Two distinctive regimes can be identified. Firstly, in the regime where the DC have a much lower probability of occurrence than the parametric photon (typically in our setup this is achieved for P > 20 mW), the CAR can be approximated by:

$$\mathrm{CAR}^* \approx \frac{N_{\text{pair}}}{N_{\text{param-param}}} \tag{5}$$

Then, in the regime of negligible DC, the CAR is mostly limited by these unpaired parametric photons. We recall that the number of true coincidences is the number of generated photons ηP^2 multiplied by the transmission efficiency (T_s, T_i):

$$N_{\text{pair}} = T_s T_i \, \eta P^2 \tag{6}$$

whereas the number of unpaired parametric photons is given by:

$$N_{\text{param-param}} = T_s(1 - T_i)\eta P^2 \times T_i(1 - T_s)\eta P^2 \tag{7a}$$

$$= T_s T_i (1 - T_s)(1 - T_i)\eta^2 P^4 \tag{7b}$$

which is the probability to generate a photon-pair while detecting one half and not detecting the other-half multiply by the probability to generate a second pair that will be detected. As it relates the detection of two unpaired signal and idler, its number scale with the power fourth as a function of power.

Thus, in the regime of negligible DC, one can write:

$$\mathrm{CAR}^* \approx \frac{1}{(1 - T_s)(1 - T_i)\eta P^2} \tag{8}$$

which decreases not really because of an intrinsic source of noise but rather a consequence of loss in the setup ($T_s < 1$ and $T_i < 1$) including detector quantum efficiencies. Figure 6.a shows a comparison between the measured CAR and simulations assuming detectors with better quantum efficiency. In theory, using superconducting SPDs, CAR as high as 10^5 could be attainable with our source.

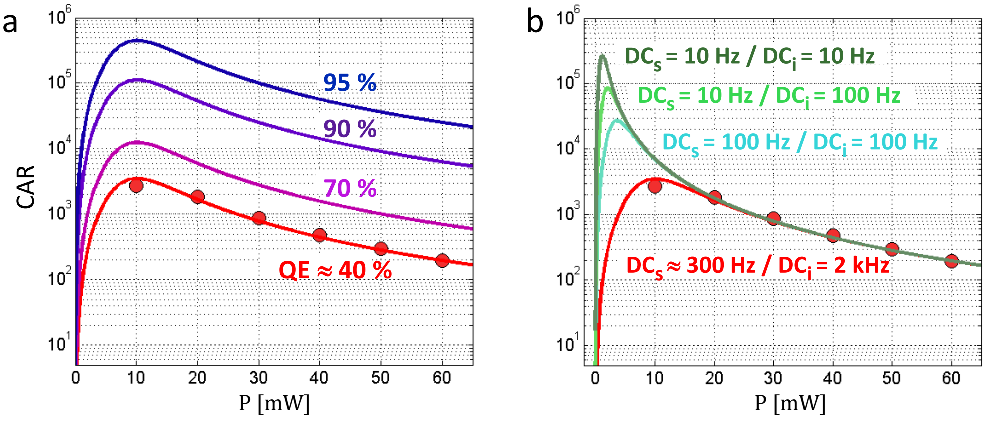

Fig. 6 Expected CAR as a function of different **a.** detector quantum efficiency and **b.** dark counts.

The second regime is at low power where the dark-counts are not negligible. As shown in Fig. 6.b, lower dark-counts shift the power for which the maximum CAR is achieved and its upper limit.
The overall detection loss and the dark-counts are currently the main limiting factor of the CAR measurement. Thus, although the CAR of our source sets the current state of the art in fiber sources, a much higher value may be obtained by replacing the detectors.

c. Alternative signal to noise ratio: In most experiments, the CAR is a good approximation of the signal to noise ratio (SNR). However, in our Raman-free configuration, one can argue that the CAR may no longer be a good approximation of the SNR, giving at best a strong underestimation. Most quantum applications temporally

post-select the coincidence events such that they are only limited by the amount of noise in the coincidence peak. Usually, when measuring the CAR, it is assumed that the measurement of the accidental peaks gives a good indication of the noise present in the coincidence peak. This assumption is rather true in presence of Raman-scattering where the noise is distributed equally in the coincidence and accidental peaks (see Fig. 7.b). However, in a Raman-free configuration, the noise contribution of the central peak is significantly lower than the accidentals (see Fig. 7.a) and thus, taking the accidentals peak as an estimation of the amount of noise may not be relevant in this case.

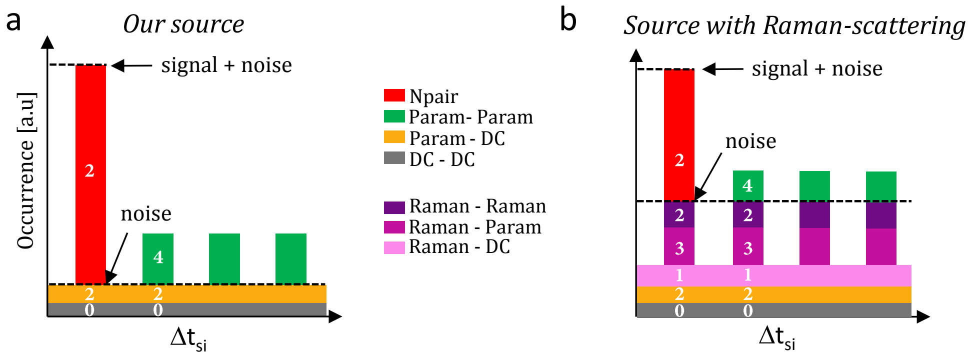

Fig. 7 Signal and noise distribution in **(a)** our fiber source and **(b)** in a standard fiber-based architecture which present Raman-scattering.

For these reasons, we propose a second evaluation of the signal to noise ratio of our source defined by the ratio of coincidences and accidentals corrected from the unpaired parametric photons:

$$SNR = \frac{N_{\text{coinc}}}{N_{\text{acc}} - N_{\text{param-param}}} \tag{9}$$

$$\tag{10}$$

In our Raman-free configuration, this simplifies to:

$$SNR = \frac{N_{\text{coinc}}}{N_{\text{unco}}} = \frac{N_{\text{pair}} + N_{\text{param-DC}} + N_{\text{DC-DC}}}{N_{\text{param-DC}} + N_{\text{DC-DC}}} \tag{11}$$

In the regime where DC-DC are negligible, one can write:

$$SNR^{*} \approx \frac{N_{\text{pair}} + N_{\text{param-DC}}}{N_{\text{param-DC}}} = \frac{N_{\text{pair}}}{N_{\text{param-DC}}} + 1 \approx \frac{N_{\text{pair}}}{N_{\text{param-DC}}} \tag{12}$$

which does not depend on the pump power since both terms share a quadratic power dependence.

Figure 8 describes the SNR of our source. At low power, the experimental SNR and the CAR exhibit the same behaviour, limited by the DC-DC coincidences. However, unlike the CAR, the SNR does not decrease with pump power but converges toward a constant of around ≈ 9000 (as expected from Eq. 12). In comparison, in a standard silica-core fiber source the SNR would decrease (as 1/P) due to the presence of Raman-parametric coincidences ($\propto P^3$) which increase faster than the true coincidences (quadratic).

[1] M. Cordier, A. Orieux, B. Debord, F. Gérôme, A. Gorse, M. Chafer, E. Diamanti, P. Delaye, F. Benabid, and I. Zaquine, Optics express **27**, 9803 (2019).
[2] B. Debord, F. Amrani, L. Vincetti, F. Gérôme, and F. Benabid, Fibers **7**, 16 (2019).
[3] M. Zeisberger and M. A. Schmidt, Scientific reports **7**, 11761 (2017).
[4] M. Zeisberger, A. Hartung, and M. Schmidt, Fibers **6**, 68 (2018).
[5] M. Cordier, Theses, Université Paris-Saclay (2019).
[6] K. Garay-Palmett, H. J. McGuinness, O. Cohen, J. S. Lundeen, R. Rangel-Rojo, A. B. U'ren, M. G. Raymer, C. J. McKinstrie, S. Radic, and I. A. Walmsley, Optics express **15**, 14870 (2007).
[7] M. Azhar, N. Joly, J. Travers, and P. S. J. Russell, Applied Physics B **112**, 457 (2013).
[8] K. Lynch-Klarup, E. Mondloch, M. Raymer, D. Arrestier, F. Gérôme, and F. Benabid, Optics express **21**, 13726 (2013).

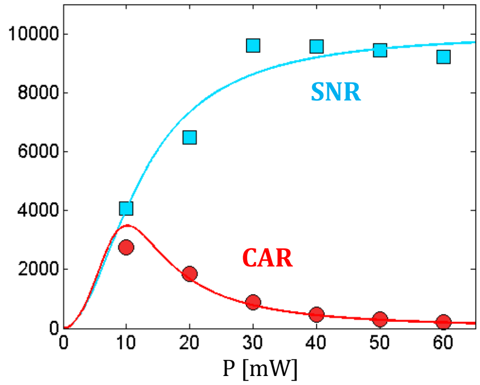

Fig. 8 Experimental CAR (red dots) and SNR (blue square). The plain lines are simulation based on polynomial fits of the coincidences, accidentals and uncorrelated.
